# Developing a Co‐Produced Practice Framework to Support Personalised Safety Planning for Adults Experiencing Suicidality

**DOI:** 10.1111/hex.70423

**Published:** 2025-09-16

**Authors:** Katherine McGleenan, Isabel Gordon, Hollie Smith, Jill Barker, Rebecca Lilley, Tara Scott, Paula Mart, Victoria Walker, Nicola Clibbens, Julie Taylor, Sarah Fishburn, Andrew Ramtohul, Sandra Chidimma Nwokoroku, Darren Flynn

**Affiliations:** ^1^ The Research and Innovation Team, Cumbria Northumberland Tyne and Wear NHS Foundation Trust, St Nicholas Hospital Newcastle upon Tyne UK; ^2^ The School of Health and Life Sciences Teesside University Middlesbrough UK; ^3^ Centre for Rehabilitation, School of Health and Life Sciences Teesside University UK; ^4^ Cumbria Northumberland Tyne and Wear NHS Foundation Trust UK; ^5^ Carlisle Eden Mind UK; ^6^ Department of Nursing, Midwifery and Health Northumbria University UK; ^7^ Institute of Health and Wellbeing University of Cumbria UK

**Keywords:** co‐production, mental health, personalisation, qualitative, safety planning, self‐harm, suicide

## Abstract

**Introduction:**

Suicide safety plans are widely used internationally in health and social care settings. This study aimed to co‐produce a framework for supporting personalised safety planning, sensitive to the needs, preferences and values of people experiencing suicidality.

**Methods:**

Phase 1 conducted semi‐structured interviews to explore the views and preferences of adults with lived experience of suicidality on the content and implementation of personalised suicide safety planning. In Phase 2, interactive practitioner workshops reviewed and refined a draft framework for personalised suicide safety planning. Data analysis was conducted in two steps: an inductive thematic analysis of interview data, followed by a deductive–inductive approach to develop the themes using data from two workshops with practitioners.

**Results:**

*n* = 11 adults with current or previous lived experience of suicidality participated in semi‐structured interviews, and *n* = 16 practitioners from primary care, secondary care, third sector and emergency services involved in providing suicide prevention were recruited to two workshops. Two overarching themes and six sub‐themes were identified: (1) the *personalisation of safety planning (*sub‐themes—*co‐production, involving family and friends, true personalisation)* and (2) the *process* of safety planning (sub‐themes—*implementation, format* and *purpose*) were used to inform the structure of a prototype personalised suicide safety planning framework.

**Conclusions:**

Personalised suicide safety planning requires early intervention and a person‐centred approach. Pivotal to this is the need to move away from standardised tools towards the development of a workforce with the skills and confidence to work flexibly and collaboratively with the people they are supporting. Future research is needed to test the utility of the framework in a range of settings, including primary care, urgent care and the third sector.

**Patient or Public Contribution:**

This study was co‐produced from the outset by people with personal experience of suicidality. Pre‐study public engagement helped inform the study design, and peer researchers on the study team collaborated in all stages of the process from design through to dissemination, including development of this manuscript. PPI involvement was included in practitioner workshops and in producing accessible dissemination materials.

## 1 Introduction

Suicide is a complex behaviour with multiple aetiological factors, some of which are poorly understood [1]. It often results from a combination of health, environmental and social risk factors, including, but not limited to, mental health conditions, traumatic experiences, substance misuse, social isolation, serious illness and stigma [1, 2, 3]. More than 700,000 people die by suicide every year worldwide [4], with 5642 suicides (10.7 deaths per 100,000 people) registered in 2022 in England and Wales [5]. With timely and appropriate intervention, suicide is considered preventable [4]. The devastating impact of suicide, on individuals, their families, friends, colleagues and communities, is a key driver for research to improve preventative interventions [6, 7]. UK health and suicide prevention strategies highlight the need for preventative approaches that are personalised and co‐produced [7, 8].

An over‐reliance on risk assessments or the identification of population risk factors may provide false reassurance by both failing to accurately identify the risk of suicidal behaviours at an individual level or identify suicide risk in ‘low risk’ population groups [9, 10]. Population‐based statistical suicide risk factors are not designed to predict individual risk of suicide at a single point in time but instead indicate lifetime risk of a whole population [1]. There are, therefore, concerns regarding the predictive validity of suicide risk assessment tools [11]. Despite these known limitations, a study of suicide risk assessments in UK mental health services found that most tools used in practice aim to predict self‐harm or suicidal behaviour and that the scores of these assessment tools routinely informed clinical decisions [12].

A study relating to how individuals who self‐harm manage their own risk reported that effective risk management involves good relationships between individuals and clinicians, which fosters a sense of ‘true collaboration’ [13]. Service users and carers report that risk‐focused approaches are unhelpful in meeting their needs and keeping them safe; they want practitioners to build a human connection and validate their distress, which instead instils hope when life feels hopeless [14]. A lack of involvement can leave people unsure about what to do in a crisis [12].

Improved experiences and outcomes related to suicide prevention have been reported when people actively shape their care and support [13, 15, 16, 17]. An individualised approach to suicide risk assessment that accounts for the specific context of the person is recommended in the current UK suicide prevention strategy and guidance for self‐harm prevention [7, 8, 17, 18].

Safety planning interventions have been identified as feasible and acceptable to adults experiencing suicidality and clinical staff [19, 20]. A suicide safety plan consists of predetermined, prioritised coping strategies for use before, during and after a suicidal crisis. It is created with the person and includes information about sources of support [16, 21] with collaboration and the therapeutic alliance described as paramount [22]. The efficacy of safety planning tested in a trial with veterans experiencing suicidality showed that veterans who completed safety plans demonstrated a 45% reduction in suicidal behaviour at 6‐month follow‐up, compared with veterans who did not form a safety plan [17]. Veterans with a safety plan were also more than twice as likely to attend mental health treatment during the 6‐month follow‐up period [17]. A systematic review concluded that safety planning was associated with reductions in suicidal behaviour, depression, hopelessness and reduced the need for hospital admission through improved treatment adherence [19]. Hospital staff reported that safety planning complemented existing services, and adults experiencing suicidality reported appreciating its simplicity and person‐centred approach [20].

Safety planning shows promise as a valuable clinical tool in healthcare settings [17]; however, without attention paid to *how* safety planning is implemented from the perspectives of service users, carers and staff, observers have noted that safety planning risks becoming an unhelpful ‘tick‐box’ exercise [23]. To address a gap in understanding about *how* personalised and collaborative safety planning ought to be delivered from these perspectives, the following research question was co‐produced with a team of researchers, practitioners and people with lived experience of suicidality: ‘What is the optimal approach to the co‐design of personalised safety plans for people experiencing suicidality?’. The study aimed to develop a prototype framework to support practitioners in delivering collaborative, personalised safety planning (PSP) for people experiencing suicidality.

## 2 Materials and Methods

### 2.1 Study Design

This study is reported according to the Consolidated Criteria for Reporting Qualitative Research (COREQ) [24] guidelines. This qualitative study was conducted in two phases. Phase 1 used semi‐structured interviews with adults with experience of suicidality to explore their views and preferences about safety planning. Using the themes developed in Phase 1, Phase 2 used interactive workshops to develop a draft PSP framework. The study was underpinned by a pragmatist epistemology [25, 26] based on the idea that knowledge is rooted in experience.

The study adopted a co‐production approach to ensure that the study findings would be sensitive to the needs, preferences and values of people experiencing suicidality. Co‐production is regarded as a way of activating the benefits of lived experience in the research process [27]. To achieve true co‐production, a shared decision‐making approach was adopted [28, 29]. It was critical that the study team comprised researchers, peer researchers and people with lived experience of suicidality from the start and throughout. To ensure all members of the study team could contribute, key principles of co‐production, as set out in NIHR guidance [29], were adopted, including the sharing of power, perspectives and skills; respecting and valuing the knowledge of all those working together on the research and reciprocity; and building and maintaining relationships. Regular meetings were held with members of the team to ensure that co‐production principles were being achieved and to ensure that the team felt supported and valued in their contribution to the research. This provided direction to ensure that there was an adequate emphasis on building and maintaining relationships, respecting and valuing each other's knowledge and that a shared understanding and ownership was achieved [29].

### 2.2 Ethical Considerations

Ethical approval was obtained from the School of Health and Life Sciences Research Ethics Sub‐Committee at Teesside University (Ref no. 6398). Potential participants were provided with a participant information sheet at least 24 h before being invited to provide consent to take part. Participants were informed of their right to withdraw at any time without giving a reason. They were also given information about how their personal data and data from the study would be managed.

Due to the potential for distress related to the research topic, and to ensure safe ethical practice when involving people with experience of suicidality, safeguarding protocol and distress protocols were co‐produced by people with lived experience and the research team [30, 31].

### 2.3 Sample and Recruitment

A purposive sampling approach was used to identify individuals with lived experience of suicidality and practitioners from different settings with experience of providing support for suicidality. In Phase 1, recruitment of people with lived experience of suicidality was supported by named recruitment gatekeepers within third‐sector organisations (voluntary or charitable) in England who distributed study information to individuals who met the eligibility criteria.

Eligibility required individuals to be aged over 18 years, have lived experience of suicidality, and be receiving support from a third‐sector organisation at the point of recruitment. Interested participants were invited to an initial meeting with a researcher by email or telephone to discuss their participation and screen for eligibility.

In Phase 2, participants were recruited from organisations across the health and social care sector with a role in supporting people experiencing suicidality via established suicide prevention networks across the North of England.

### 2.4 Data Collection

Phase 1 semi‐structured interviews were conducted face‐to‐face, via telephone or via Microsoft Teams, depending on the participants' preference. Two people were present at each interview, one researcher and one study team member with expertise in supporting people with their mental health. After discussion with the research team, including those with lived experience of suicidality, the decision was made that a minimum of demographic data were to be collected to establish eligibility from participants to preserve their anonymity, to build a trusting rapport and to ensure that the participant felt comfortable sharing their experiences.

The interview topic guide was developed using published safety planning evidence and was co‐produced. It included questions to explore participants' views about how practitioners could best support people when managing risk and developing safety plans, including their form and content. Interviews concluded with a debrief and well‐being check. Field notes were taken during interviews to aid analysis and to enable reflexivity (i.e., a critical examination of the researchers' perspectives, assumptions and potential biases throughout the research process [32]). Interviews were audio recorded and transcribed verbatim by an external transcriber.

In Phase 2, two 3‐h interactive workshops were facilitated by two or three researchers. Before the workshops, the themes from Phase 1 were compiled into a draft PSP framework. During the workshop, the draft PSP framework was shared with participants to refine and co‐create a prototype version and develop guidance for its use. The workshop discussions were generative, bringing together the experiences of those who have used safety plans and those who have supported people with their suicidality. During these discussions, workshop participants explored the benefits of the PSP framework, as well as barriers and enablers to adoption of the framework. The workshops were audio recorded, with field notes taken to aid reflexive analysis and transcribed verbatim by the study team.

### 2.5 Data Analysis

Phase 1 interview transcripts were analysed using a six‐step inductive, reflexive thematic analysis (TA), whilst acknowledging that analysis of qualitative data is neither a wholly inductive nor deductive process, rather a ‘hybrid’ of the two [33, 34] as outlined by Braun and Clarke (2019) [32]. Reflexive TA involves six stages: (1) dataset familiarisation; (2) data coding; (3) initial theme generation; (4) theme development and review; (5) theme refining, defining and naming; and (6) writing up.

Stage 1: All transcripts, along with field notes, were read in detail by two reviewers to facilitate understanding of the dataset.

Stage 2: All transcripts were coded independently by two researchers to initiate an in‐depth understanding of the experiences and views of participants [24, 35]. Coding was conducted using the software package NVivo (Lumivero; v12.7.0). Initial codes were shared with the study team and discussed to ensure firstly that the codes were co‐produced from academic and lived experience perspectives, and secondly that the research team reflected on the codes using field notes and reflections from team members to explore the impact of their own biases and experiences on the analysis.

Stage 3: After initial coding, the researchers met to develop themes and sub‐themes through an iterative process moving between codes and transcripts paying attention to team reflections to ensure the positionality of the researchers was considered throughout the process [36, 37].

Stage 4: Through discussion, the research team reviewed the analytic process to ensure that the themes represented the data from participants, were contextually meaningful, and made sense in relation to direct quotes from the data and the dataset as a whole [38].

Stage 5: During this stage, themes were defined and given names that represent the views obtained from the participants. This process was iterative and involved input from all members of the research team (academic and lived experience) to ensure that the theme names were representative of the data gathered but also meaningful to the reader.

After completion of stage 5, a visual representation of the relationships between the themes was created. This formed the draft framework for further development in Phase 2 workshops.

Phase 2 workshop transcripts were analysed using a deductive–inductive reflexive TA approach [32, 33, 34, 35, 36, 37]. Firstly, the themes and sub‐themes within the draft framework were used to deductively code the workshop data. This was carried out independently by two researchers. Codes were then presented to the research team for discussion and to enable identification of convergence and divergence in the data. In a second analytic step, to ensure all data from the workshops had been coded, an inductive approach was used to add new codes and identify any new themes or sub‐themes following the analytic stages outlined in phase 1 [32, 33, 34, 35, 36, 37].

## 3 Results

### 3.1 Phase 1: Individual Interviews

Eleven individuals aged between 24 and 62 years, 2 male, 9 female, took part in an interview. All participants reported experiences of suicidality for which they had received support from either family, friends, third sector or health services. Analysis resulted in two themes, each with three sub‐themes shown in Table 1. The results are presented by theme, including the sub‐themes and illustrative quotes from participants.

**Table 1:** Themes and sub‐themes.

Theme 1: Personalisation	Theme 2: Process
Sub‐themes:	Sub‐themes:
1a: Meaningful co‐production	2a: Having a shared understanding of the purpose of the plan
1b: Supporting and involving family and friends	2b: Format of the plan
1c: Making the plan truly personal	2c: Implementation of the plan

### 3.2 Theme 1: Personalisation

The theme of personalisation refers to the importance participants placed on recognition of their individual needs and preferences. Experiences were often described as neither personalised nor co‐produced; instead, interventions were more task‐orientated and impersonal. The use of generic safety planning templates was experienced negatively. Positive experiences related to feeling listened to, and when safety planning was individualised. Three sub‐themes provide details of the participants' experiences of and preferences for personalisation.

#### 3.2.1 Sub‐Theme 1a—Meaningful Co‐Production

Participants described their experiences of safety planning as typically led or directed by a practitioner following a pre‐formatted document, rather than their preferred approach where the practitioner guided and supported them to develop their own plan.

‘It would definitely have been better if they'd have sat down with me and I'd have been able to write it out with them, rather than them impose their own thing on me’.

Participant 2

The interpersonal process of engagement to enable safety planning was key to personalisation of safety plans for participants, who wanted the process to be about the person, rather than focused on clinical processes.

‘…not a very sort of person centred or personal approach in the slightest … it was very clinical’.

Participant 9

Participants described their most positive experiences were when practitioners had the skills and confidence to actively listen to them.

‘…I was aware at the time of the … professionals who … had the skills and the confidence to be able to listen to what I said and accept it and treat me as an individual’.

Participant 4

When participants were not involved in developing the plan, they felt that they did not have ownership of it. Importantly, meaningfully co‐producing a safety plan relied on interpersonal engagement described as ‘*…that human touch … it is about understanding you as a person’* (Participant 9), which takes time, sometimes requiring multiple discussions. Participants wanted their safety plan to be realistic and useful, which they believed to be best achieved through collaboration.

‘…it was collaborative and … time was given. It was revisited, it was realistic, it had my voice, it was taken from the point of view of a problem‐solving approach in the sense that it wasn't complicated’.

Participant 5

‘I always find that just being friendly, open and approachable is the best way’.

Participant 7

#### 3.2.2 Sub‐Theme 1b—Supporting and Involving Family and Friends

Most participants wanted family and/or friends to be involved; however, they were clear that this needed to be discussed and agreed on individually. Some people may not have, or may not wish to involve, family or friends, or they may not be available to support them. Alternative support, in the absence of family or friends, such as peer support, was suggested. Where family and/or friends are involved, participants did not want their involvement to be tokenistic; instead, participants wanted them to be involved throughout.

‘If you're gonna have a partner or a trusted other involved in safety planning it can't be just at the end, “Here, here's the bit of paper.”’

Participant 5

Importantly, participants were concerned for the well‐being and support needs of family and/or friends.

‘You always have to have that thought in your head about their mental health as well’.

Participant 7

Participants stated that there should be support for family and/or friends who often take the burden of responsibility, and associated stress, for providing support during a suicidal crisis and can play a key role in helping the person activate their safety plan.

‘I suppose knowing that they had their own support and their own safety plan in a way helps. Peer support because you can share in that environment without the guilt of what it does to your family. Some sort of peer support or support network for, yes, for family’.

Participant 4

#### 3.2.3 Sub‐Theme 1c—Making the Safety Plan Truly Personal

Participants described experiencing generic approaches to safety planning as unhelpful and emphasised the need to make safety planning *truly* personal. Participants wanted practitioners to allow creativity, engage, listen and ‘*hear their voice’* (Participant 5). Participants believed that no matter ‘*…how much in crisis’* (Participant 5) they were, they wanted their safety plan to be developed collaboratively. Participants described safety planning that included using their creativity to identify the most meaningful elements of staying safe for the individual.

‘I've got post‐It notes. Little random words, like “smile” or “watch a TV Show” or “go and see a friend.”’

Participant 10

‘I think with the safety planning drawing, writing, just having that space to be creative can really help’.

Participant 6

### 3.3 Theme 2: Process

This theme refers to the practical aspects of how a suicide safety plan is developed. Interpersonal processes were key and included the tone and content of the discussion. Without interpersonal engagement, the process of safety planning is reduced to:

‘…just words on a page, you know … sent over in an email’.

Participant 8

The process of developing safety plans also required mutual agreement about the purpose of the safety plan, its content and design, including understanding why certain elements are important, the process to activate the use of the plan by the person and those around them, and how the plan would be reviewed.

#### 3.3.1 Sub‐Theme 2a—Having a Shared Understanding of the Purpose

Participants talked about their perception that there may not always be a shared understanding between staff and those in crisis about the purpose of safety planning. Some participants described staff as ‘risk’ rather than ‘safety’ focused, and their belief that this relates to staff meeting an organisational need for assurance.

‘It felt like from their point of view they had a safety plan, but I didn't necessarily have anything that made me feel any safer’.

Participant 4

‘I felt that it was to cover their backs. I don't think I've really had a safety plan put together that I've felt has been for my benefit’.

Participant 8

Participants believed that a personalised process of developing a safety plan helped them explore different, accessible and useful ways to cope. They wanted the safety plan to be ‘*simple and practical*’ (Participant 5). A lack of a shared understanding limited safety planning to the immediate crisis, which was contrary to the preferences of participants who wanted to focus on the longer‐term quality of life.

‘The crisis part, for me … meets an immediate need to keep the person alive. Later, the more sophisticated safety planning … can involve much more important things’.

Participant 5

When safety planning included a future focus, participants described feeling more hopeful and able to identify a reason to live, ‘*to thrive not just survive*’ (Participant 11).

#### 3.3.2 Sub‐Theme 2b—The Format of the Plan

Participants understood that safety plans would, even when personalised, contain some core features, such as a predetermined list of sources of support. However, meaningful involvement from the individual ensures the safety plan is in a useable format and contains both the core features, but also the ingredients that the person can relate to. Without this, the safety planning *process* was described as unhelpful.

‘…they give you a generic plan which doesn't pertain to you, it often contains parts which are not relevant to you which is churned out basically to meet a [system quality] criteria but doesn't actually have any meaning to the individual…’

Participant 1

There was variation in what participants found to be the most helpful format. For some, written formats were unhelpful ‘*For me, writing it down never works*’ (Participant 10) and others found detailed writing the most helpful. Electronic versions were helpful for some participants, including through portable technology.

‘I'm a bit of an obsessive planner and because I feel like nothing happens unless I write it down’.

Participant 9

‘Well, I've got a template on my memory stick, and I also have one on my mobile phone’.

Participant 11

#### 3.3.3 Sub‐Theme 2c—Implementation of Safety Planning

Implementation was used by participants as a description of both the development, review and ongoing use of safety plans, seen as a ‘pathway’ from initiation through to ongoing maintenance rather than as a ‘one‐off’ intervention. This included who was best placed to support safety planning, when they were initiated and the process for reviewing, updating and sharing.

Participants described the importance of having the right support person; this related to the different skills, experience and qualifications of the person. Neither having a specific professional qualification nor having lived experience was seen as essential. Most important to participants was the interpersonal skills of the person, particularly taking a non‐judgemental approach, showing compassion and using active listening skills.

‘I think if you've got someone who is therapeutically skilled, it doesn't have to be a qualified person, just someone with the right kind of skills to actually be able to sit down and reflect with you and ask the right questions’.

Participant 11

In relation to the timing of the initiation of a safety plan, most people reported them being initiated around the time of a suicidal crisis. Whilst participants understood that initiating a safety plan during a crisis had some logic, they felt this was not the *best* time for the plan to be developed.

‘I think plans that are done while you're either just coming out of a crisis or just getting to that point of crisis that I'm sure they have value’.

Participant 9

Participants felt they would be more able to collaborate and understand what they wanted from their safety plan at a time when they were less distressed. During a period of crisis, participants described being less able to ‘*have the mental space to sit and actually think about a plan’* (Participant 9). It was also important that people felt able to make informed decisions about the content of the plan which may be difficult during a crisis.

‘…get the person in a good place to even be able to make informed decisions’.

Participant 8

Participants referred to ‘*doing groundwork*’ (Participant 9) for a safety plan with or without the immediate presence of suicidal crises, suggesting that safety planning requires an iterative process.

Whilst participants experienced safety planning as most often a one‐off event without follow‐up, the ongoing review and update of safety plans was considered important. This was because safety plans need to be contextually relevant to individual circumstances, needs and preferences, which may change over time. What was clear is that a follow‐up should be planned with the individual.

‘…initially it felt quite supportive … however then … nothing happened afterwards. There was no follow up’.

Participant 6

‘Actually agree that time with the individual and say you know, “I'd like to check in with you but what would be suitable for you?” And “what would the method, face to face, or MS Teams or just over the phone?” And just, yes, just check progress, check how they're feeling, what they've done, has it worked for them? If it hasn't worked let's evaluate and put other things in place. What their support network is like. Has it been useful?’

Participant 6

There was general acknowledgement that safety plans should be shared with professionals and the person's support network. However, there was no consensus amongst participants about how this should happen, when or with whom. There was particular concern that decisions to share safety plans with family and/or friends were made in collaboration with the individual.

‘There has to be a sharing of information … nuanced as the person's life changes’.

Participant 5

### 3.4 Phase 2: Practitioner Workshops

**Figure 1:**
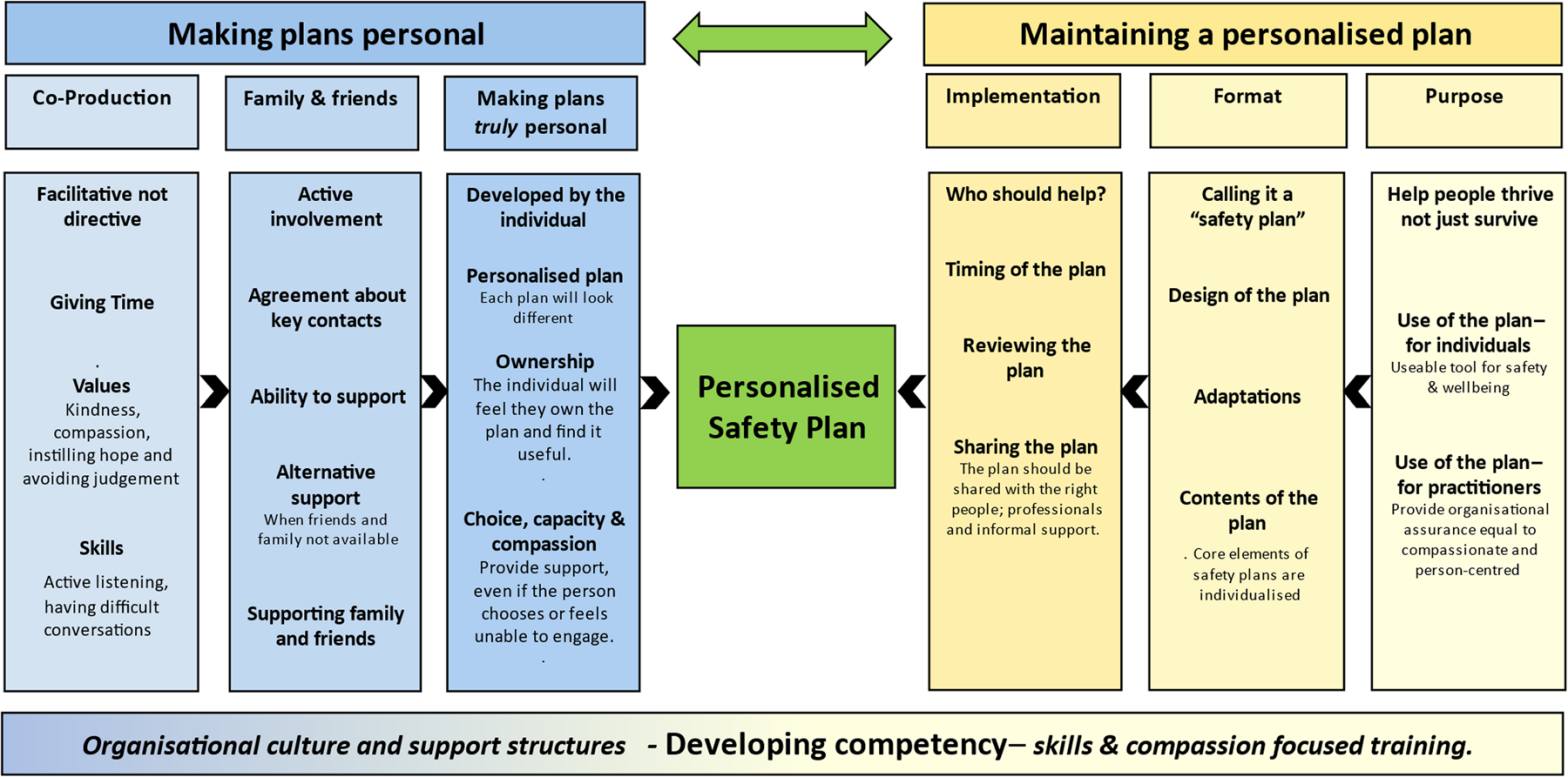
Prototype co‐produced personalised safety planning framework.

Using the themes identified in Phase 1, a thematic map was developed to illustrate and summarise the themes in readiness for two interactive practitioner workshops. A total of 16 participants took part in one of two workshops. Workshop participants were recruited from two NHS mental health trusts in England, primary and social care, third‐sector organisations and emergency services, shown in Table 2. Following the synthesis of data from interviews and workshops, a prototype PSP framework was developed, shown in Figure 1.

**Table 2:** Workshop participant expertise.

Expertise of workshop participants	Number
Autism clinical lead	1
Lived experience inclusion lead	1
Mental health nurses	4
Experts by experience	2
General practitioner	1
Third‐sector provider practitioners	2
Social worker	1
Police officers	2
Psychiatrist	1
Mental health crisis service manager	1

The findings from the workshops are summarised with an overview of the generative discussion about the framework, followed by a summary of the identified barriers and enablers to implementation.

### 3.5 Perspectives on the Draft PSP Framework

Workshop participants identified several benefits of having a personalisation framework to guide practice. Having such a framework was felt to emphasise the need for personalisation rather than adoption of a ‘one size fits all’ approach. Having the framework as guidance was also believed to prompt more proactive preventative approaches rather than one‐off crisis responses. The framework was described as a tool to facilitate a trauma‐informed (rather than diagnosis‐led) way of working. This may in turn help practitioners balance a personalised approach with the need for risk assessment often required to provide organisational assurance. Importantly, the framework was believed to align with current practice.

### 3.6 Enablers

As people experiencing a suicidal crisis can be in contact with different staff across a range of agencies, including acute and non‐acute settings, participants felt that the framework should be implemented alongside multi‐agency training. This in turn could optimise its use as a preventative intervention and help facilitate planned follow‐up.

A focus on organisational culture and the values of the workforce was felt to be key to implementation. From the perspective of personalisation, people with lived experience emphasised the need for staff in different agencies to all be ‘*singing from the same hymnbook*’. When safety plans were available electronically with the addition of clickable links to further information, this optimised implementation of cross‐agency information sharing. Most important to implementation at an individual level was that the person themselves had ownership of their safety plan and had access to it in whatever format worked for them.

### 3.7 Barriers

Participants felt that the health and social care organisational culture is too often focused on risk and that current systems of surveillance drive the need for staff to provide organisational assurance. Without a change that allows the workforce to invest in more personalised approaches, adoption of the personalisation framework may be slow. Due to resource pressures, it was felt that practitioners are more likely to resort to tick box processes because working in more personalised ways takes more time and may require more than one session. Further to this was the lack of resources or a system to prompt follow‐up to safety plans. Whilst the TA suggested that this is determined by individual need and context, without a system to support follow‐up in practice, it may easily be missed.

## 4 Discussion

In recognition of a gap in the suicide prevention evidence, the focus is on how suicide safety planning can be delivered in personalised and collaborative ways. The findings of this novel qualitative reflexive TA and co‐production study illustrate that, for safety plans to be meaningful and effective, personalisation and collaboration in the development and use of safety plans are important for people experiencing suicidality. The findings here add to existing evidence suggesting that safety planning is a valuable clinical intervention in healthcare settings [17]. By developing a framework and guidance for practitioners, which focuses specifically on the personalisation of safety planning, rather than the safety planning tool itself, practitioners will be supported to prioritise what is known to be most effective [8].

The findings here support previous evidence that improved experiences and outcomes related to suicide prevention are reported when people actively shape their care [13, 17]. The prototype PSP framework emphasises that a positive and understanding approach to safety planning helps to build a therapeutic alliance. Not only does this improve the experience of safety planning, but it can also provide a protective factor against suicide [39]. The experiences of participants in this study underline the negative impact of not getting this right. The main skill deficit reported by participants when referring to their experiences with practitioners was that they were not really listening. Active listening plays an important role in people feeling heard and sufficiently valued by others [40]. A failure to utilise these skills can lead to individuals feeling unimportant and their experiences being dismissed [41]. The prototype PSP framework focuses on the interactions between the practitioner and the individual, and the context, skills and values needed to optimise personalisation of the safety planning process, human interaction and the importance of actively listening.

One barrier to the implementation of the framework was the need for practitioners to balance organisational assurance with the PSP processes. The NHS long‐term plan [8] aimed to give people more control over their health and care, including personalised care, supported self‐management and shared decision making. Aligned to the PSP framework developed here is the NHS England guide to support integrated care systems (ICSs) to understand and create the conditions for sustainable implementation of personalised care [42].

A critically important finding of this study was the perception of individuals that practitioners did not have the time or skills required to meet their needs. This may suggest an unmet training need or, perhaps more concerningly, a lack of sufficient resources to move suicide prevention forward from the unhelpful tick box risk assessments identified previously [23] and reiterated in the findings here. To address this, training interventions and initiatives to aid practitioners in developing the necessary skills required to support PSP are crucial. Similarly, the development and implementation of specific resources (such as the prototype PSP framework developed by this study) would ensure that practitioners have the necessary resources in place to support them to move away from tick box assessments in a way that balances the need for organisational assurance with the needs of the individuals.

Whilst practitioners reported positively on the prototype PSP framework, they expressed concerns about the organisational culture, structures and processes which they felt presently hindered their ability to operationalise a personalised approach. Workshop participants also felt constrained by organisational systems and issues of governance. A focus on the implementation across different contexts, using a ‘system thinking’ approach, is needed to consider the influence system constraints may have on embedding PSP in practice [43]. An individualised approach to suicide prevention is advocated in the current UK suicide prevention strategy and clinical guidance for self‐harm prevention [7, 18]. Our findings resonate with this approach and reinforce the national priority for more research in this area, to improve the evidence for effective interventions [7] and to optimise implementation in a range of settings where suicidal people are supported, as advocated by the participants here.

### 4.1 Strengths and Limitations

This study has taken a novel approach to explore what is needed to implement collaborative and personalised suicide safety planning. By including co‐production, the prototype PSP framework reflects the views of people with lived experience of suicidality and those who provide safety planning interventions throughout the research process. The study's approach was recognised as good practice, receiving a patient and public involvement award [44]. The small sample size enabled deeper exploration of the experiences of individuals but may limit the transferability of the findings. An important limitation of this study is the lack of diversity within the sample, partly related to the small sample size, but also related to the limited geographic location of the participants and their experiences with safety planning. Future research should ensure sample diversity with particular attention to underserved groups. It is also important to acknowledge that all of the participants in this study were actively engaged with mental health services. This, therefore, may limit the generalisability of the prototype PSP framework to individuals who are not engaged with such services. Future work should seek to obtain the views of those not engaging with services.

## 5 Conclusion

This study has demonstrated that rather than adopting risk‐focused suicide prevention practices, safety planning is preferred and optimal when personalised. The findings here suggest that, in line with previous evidence, there is a need to move away from one‐off standard tools that are limited to crisis situations towards the development of a workforce with the resources, skills and confidence to work in more personalised and collaborative ways. The prototype PSP framework developed in this study provides a first step in the development of guidance for practitioners in the process of personalising safety planning.

There is scope for future research to further refine and explore the utility of the framework and guidelines across larger, more diverse populations and across a range of regions and settings. Moreover, future research is needed to explore and develop the implementation guidance with a specific focus on overcoming barriers and enhancing enablers. We recommend that future research in this area continue to include co‐production.

## Author Contributions

**Katherine McGleenan:** conceptualization, data collection & analysis, writing original draft – review and editing. **Isabel Gordon:** methodology, data collection and analysis, writing original draft – review and editing. **Hollie Smith:** data collection & analysis, writing – review and editing. **Jill Barker:** methodology, data collection and analysis, review and editing. **Rebecca Lilley:** data collection and analysis, writing – review and editing. **Tara Scott:** conceptualization, data collection and analysis – review and editing. **Paula Mart:** conceptualization, data analysis, review and editing. **Victoria Walker:** data analysis, review and editing. **Nicola Clibbens:** writing, review and editing. **Julie Taylor:** methodology, writing, review and editing. **Sarah Fishburn:** methodology, data collection, review and editing. **Andrew Ramtohul:** methodology, review and editing. **Sandra Chidimma Nwokoroku:** data analysis, review and editing. **Darren Flynn:** conceptualization, methodology, data collection and analysis writing original draft – review and editing.

## Ethics Statement

School of Health and Life Sciences Research Ethics Sub‐Committee at Teesside University (Ref no. 6398).

## Conflicts of Interest

The authors declare no conflicts of interest.

## Data Availability

Data are available upon request from the corresponding author.
